# Psychopathological phenotypes in childhood migraine

**DOI:** 10.1186/1129-2377-16-S1-A31

**Published:** 2015-09-28

**Authors:** Maria Esposito, Marco Carotenuto

**Affiliations:** Dipartimento di Salute Mentale e Fisica e Medicina Preventiva, Seconda Università degli Studi di Napoli, Naples, Italy

Migraine headache represents a frequent reason for neurological evaluation in developmental age. Many studies in the last decades have been focused on the role of psychopathological profiles among migraineur children. On the other hand, conflicting results have been reported about the different psychopathological profiles among subjects affected by migraine.

The role of psychopathological aspects on clinical findings among children may be relevant particularly for severity and frequency of attacks.

A recent review showed the higher prevalence in migraine children of psychological symptoms, detected by using the Child Behavior Checklist (CBCL), than healthy controls[1].

Moreover, clinical and population-based studies suggest that children with migraine are more likely to have internalizing symptoms (i.e., particularly anxiety and depression traits), as well as psychological comorbidities[1].

Nevertheless it is still a matter of controversy whether children with migraine have specific psychological vulnerabilities or if they only cope differently with stressful situations. In 2015 Arruda et al reported that children with migraine are more likely to present emotional symptoms, conduct problems, hyperactivity, peer problems, and total difficulties in psychosocial adjustment stressing the role of psychological adjustment styles as predisposing factors for developing psychopathological troubles among migraine children[2].

In this perspective we could speculate that the psychopathological profile of migraine children could be influenced by environmental or familiar elements such as, by specific psychological vulnerabilities of migraine children[2].

In this light, some studies pinpointed the role of parenting styles effects on migraine severity and frequency in children, related to parental stress levels, while a preliminary study indicated the potential value of maternal personality assessment for better comprehension and clinical management of children affected by migraine[3, 4].

According to the analysis of specific psychopathological vulnerabilities in migraine children, a higher prevalence among migraineur children of the avoidant attachment style (type A) and the significantly lower prevalence of the secure style attachment (type B) than controls was found[5]. Moreover, also significant differences among temperamental characteristics in MwA children respect to the comparisons[6], suggesting that the study of psychiatric comorbidities in pediatric headache may be enriched by these new aspects.
